# Kindergarten: Producer or Reducer of Inequality Regarding Physical Activity Levels of Preschool Children

**DOI:** 10.3389/fpubh.2018.00361

**Published:** 2018-12-07

**Authors:** Karin Oddbjørg Kippe, Pål Arild Lagestad

**Affiliations:** The Faculty of Education and Arts, Nord University, Bodø, Norway

**Keywords:** preschool children, physical activity, kindergarten, income, education

## Abstract

Several studies have shown that children have sub-optimal physical activity levels. Since preschool children (4–6 years-old) spend most of their time awake in kindergarten on weekdays, physical activity level at kindergarten is crucial. The study examines preschool children's physical activity level at kindergarten. Preschool children's activity level at kindergarten is also investigated related to activity level at leisure, gender, and mothers' education level, income, and age. Two hundred and forty four children (125 boys and 119 girls) supplied valid accelerometer data, and mothers' education level, income, and age were measured using a questionnaire. One-way ANOVA and linear regression were utilized as statistical analyses. The results demonstrated that physical activity level during kindergarten is the main contributor to preschool children's physical activity level on weekdays. Furthermore, boys were more active than girls, and preschool children's physical activity level at both leisure and at kindergarten were not associated with mothers' age, education level, or income. However, a positive association was found between physical activity level at leisure and physical activity level at kindergarten, in which physical activity level at kindergarten increases when physical activity level at leisure increases. Physical activity level was also significantly different between kindergartens. The study indicated that kindergartens increase inequality according to physical activity level among preschool children—contributing to creating differences according to low-active and high-active children.

## Introduction

Physical activity reduces the risk of illness, improves quality of life, and increases functional ability (1). Moreover, physical activity strengthens muscles and the skeleton, develops skills, reduces anxiety and depression, bolsters self-confidence, and contributes to social interaction (2). It is recommended that children engage in physical activity for a minimum of 60 min each day with moderate or high intensity (MVPA) (3). Earlier research has shown, however, that not all children satisfy these health recommendations, and that the physical activity levels that are too low (4–9). While Berglind et al. (10) found that 32.6 % of the Swedish 4-year olds fulfilled the health recommendations of physical activity, Andersen et al. (11) show that almost 60% of the children (average 3, 7 years old), fulfilled these recommendations. Kolle et al. (12) showed that respectively 95.7 and 87% of the Norwegian 6 years old boys and girls fulfilled these recommendations. Several studies have also reported that boys are more active than girls (7, 9, 10, 13–18).

Studies have revealed that, in Norway and other countries, the activity level of children declines with increasing age (8, 12, 18–20). Cooper et al. (19) found that total physical activity level decreased by 3.7% for boys and 4.6% for girls annually from the age of five. Another study reported that, from the age of six, time spent being inactive increases on average by 17 min for each year up to the age of 15 (1).

Almost all Norwegian children from 3- to 6-years-old, are in kindergarten most of their waking hours (21), and the kindergarten staff will have a major influence on children's physical activity levels. Consequently, it is asserted that the kindergarten can contribute to influencing children's health in the short and long term, and also to leveling social differences, which constitutes an important principle underlying public-health work (1). Children in Norwegian kindergartens spend a lot of time outdoors, and most of the children have access to natural areas and large areas to move on during kindergarten time. A study showed that children in Norwegian kindergartens spent approximately nearly 4 h outdoors (22). According to Herrington and Brussoni (23) outdoor activity, particularly in natural play spaces, boosts children's physical activity. Finn et al. (14) found that more than 50% of the average daily activity occurred during children's preschool hours. This demonstrates that kindergartens may be an especially important arena for decreasing social differences due to physical activity level among children. According to both Rossem et al. (24), and Borraccino et al. (25), lifestyle behavior will follow the same trend from kindergarten age up to adulthood. Several researchers highlight the importance of kindergarten staff being involved and making efforts to promote children's physical activity, and furthermore, that policy and practice in kindergarten greatly impact the total physical activity level of children (7, 26–28). Finn et al. (14) reported that the kindergarten was the strongest determinant for physical activity among children.

One of society's major challenges is to maintain the activity level of children and to prevent the development of differences in activity levels based on socio-economic variables (1). Socio-economic variables appear to exert a major impact on the activity levels of both youths and adults (29, 30). Studies find slightly divergent results, however, concerning the importance of socio-economic status, such as income and education, for children's physical activity. Cotrell et al. (31) found that children (aged 5 to 15) from families with lower incomes, received more approbation for being physically active outdoors, and their parents more often participated in the activity with them. Kimbro et al. (32) determined that preschool children from families with lower socio-economic status had more unstructured time, which contributed to more physical activity than it did for children from families with higher socio-economic status. On the other hand, Pate et al. (7) reported little difference in activity level (MVPA) between children aged three to five, considered in relation to parental education. This is supported by Telford et al. (33), which did not find a link between the physical activity of 5- to 6-year-olds in their free time, and socio-economic status.

The previous discussion points to the importance of kindergartens in relation to preschool children's activity level and reducing inequality in physical activity level among preschool children. However, there is a lack of research that has examined children's activity level in kindergarten with objective measures, such as accelerometers, and controlling for variables, such as gender, and mothers' education level, income, and age. The main aim of the current study was to elucidate whether the kindergartens succeeded to reduce inequality in physical activity level among preschool children (4–6 years-old) at leisure, by providing all preschool children with the same activity level at preschool. The purpose of the study is operationalized into the following four research questions:

1. What is the contribution of preschool children's MVPA at kindergarten in order to achieve the international health recommendation of 60 MVPA daily?
2. Are there any differences between MVPA at kindergarten in different kindergartens?
3. To what extent is preschool children's MVPA at kindergarten related to gender, and their mothers' education level, income, and age.
4. Do the kindergartens succeed to create high levels of MVPA at kindergarten, especially for preschool children with a low activity level at leisure?

## Materials and Methods

To answer the above research questions, accelerometers were used among preschool children and kindergarten staff, and questionnaires among the children's parents. Accelerometers were chosen because they can detect intensity, frequency, and duration of children's physical activity (12, 34–36). Moreover, the use of accelerometers make it possible to compare data with a national population study of physical activity level among pre-schoolers (12). Questionnaires are the most common tool for measuring education level, income, and age.

### Subjects

Of 122 preschools in four counsils in Nord-Troendelag county, 13 preschools were randomly selected to participate in the study, independently of size, and type of kindergarten. The kindergartens were located in the same socioeconomic area. A condition for participating in the study was that children were full-time in preschool. The 13 kindergartens included 364 full-time children at the age of 4–6 years. Two hundred and forty four children (125 boys and 119 girls) had valid accelerometer data, constituting a response rate of 67%. The number of 4–6 year-old children in full-time kindergarten varied widely (see Table 1).

**Table 1:** Descriptive data of the 244 children (4–6 years-old) by increasing MVPA at kindergarten.

**Kindergarten number**	**Number of children**
1	10
2	5
3	34
4	25
5	14
6	6
7	27
8	29
9	16
10	28
11	16
12	24
13	10

### Procedures

Accelerometer data and questionnaire data were collected during May and June, 2017. Prior to signing the written consent form and the data collection, preschool teachers and parents received written and oral information about the procedures and ethical standards for testing related to sports science. Actigraph GT1M accelerometers (ActiGraph, Fort Walton Beach, FL, U.S.A.) were utilized to objectively measure preschool teachers and 4–6 year-olds' physical activity over seven consecutive days, which is recommended by several researchers (12, 17, 37, 38). Participants were instructed that the accelerometer had to be placed on the right hip, which is recommended by Kolle et al. (12), and worn every day except for during sleep, showering, or other activities involving water. During the data collection, the participants (i.e., their mother and father) received an SMS each morning, reminding them to have their child wear the accelerometer. Raw data output produced from the accelerometers are expressed as counts per minute (CPM), which refers to all acceleration to which the accelerometer has been exposed, divided by the number of minutes the accelerometer has been used (12). According to the test protocol of Kolle et al. (12), counts are summed during 10 s intervals in order to capture as precise data as possible. Furthermore, the accelerometer data were classified as sedentary, light, moderate and vigorous physical activity, according to the divisions used in a national population study of physical activity level among pre-schoolers (12). According to international health recommendations, moderate and vigorous physical activity (MVPA) per day is the most relevant and used measure of physical activity level. The children's MVPA level during the time in kindergarten is also used as the dependent variable in this study.

For initializing the accelerometers, to download accelerometer data, and to validate and create accelerometer data (MVPA), Actilife v6.13.3 (ActiGraph, LLC, Pensacola, FL, U.S.A.) was used. Accelerometers were set to start recording at 6:00 a.m., the day after they were distributed and put on, in an effort to counteract the Hawthorne Effect (39). According to the test protocol, at least 480 min of daily recorded activity were required to obtain a valid day, and 20 min or more with consecutive zero counts were interpreted as non-wear time and removed (12). Furthermore, the preschool children were required to have at least two valid days to be included in the study. Data between 12:00–5:59 a.m., were excluded due to instructions concerning no accelerometer-wearing during sleep. Finally, the MVPA among preschool children at kindergarten (school day) was categorized as 8:00 a.m., −3:29 p.m., and MVPA among preschool children at leisure on weekdays was categorized as 6:00 −7:59 a.m., and 3:30–11:59 p.m. Weekend was categorized as 6:00–11:59 a.m., Saturday and Sunday. These operationalisations were made according to feedback from several of the preschool staff and parents of the preschool children, who identified these times as time spent in kindergarten and leisure, respectively.

The questionnaire was designed on the basis of already validated and reliability-tested questions from studies of Hansen et al. (40) and HUNT3 (41). The questionnaire was pre-tested by 10 parents of 4–6 year-old pre-schoolers in a kindergarten that was not selected for the study.

To visualize the importance of kindergarten according to preschool children's MVPA level at kindergarten, MVPA level among preschool children at kindergarten was categorized from 1 to 13, with the kindergarten with the highest MVPA level first (1) and by decreasing activity level until the kindergarten with the lowest activity level (13). The distribution of children in the kindergartens is presented in Table 1.

### Statistics

The distribution of the dependent variable (MVPA at kindergarten) seemed to follow a normality curve. However, the Kolmogorov-Smirnov test and the Levene's test (42) showed that the assumption of normality and similar variances was not met (*p* < 0.05). According to Vincent and Weir (43), however, the *F* test (ANOVA) produces valid results even when the sample is not normally distributed or with variability in the sample. This assertion is also supported by Lumley et al. (44), especially related to the high numbers of subjects in the present study. Lumley et al. (44) also make this point regarding the use of linear regression. A one-way ANOVA was employed to determine if there were any differences in preschool children's MVPA at kindergarten between the kindergartens, with a *post hoc* test using Bonferroni corrections. Paired sample *t*-tests were used to examine differences between children's MVPA level at leisure and kindergarten. To find to what extent preschool children's MVPA at kindergarten was related to MVPA at leisure and their mothers' education level, income, and age, linear regression was utilized. However, since the assumptions of continuous variables were not met according to mothers' education level and income (see Table 2), these variables were dichotomized into categorical variables (low education [primary school and high school], high education [university education], and low income [<500,000 nkr], and high income [500,000 nkr or more]). The level for significance was set at *p* < 0.05. Statistical analysis was performed with SPSS, version 24.0 (IBM, Armonk, NY, U.S.A.).

**Table 2:** Characteristics of the children's MVPA level at leisure and kindergarten on weekdays, according to the independent variables: Mothers' education level, income, and age.

**Mothers' education level**	**Leisure time Mean (SD)**	**Kindergarten time Mean (SD)**	**N**
Less than 7 years at primary school	21.2	71.4	2
7–10 years at primary school	33.5	48.7^*^	8
High school, vocational subjects	33.2	55.7^*^	46
High school, specialization in general studies	33.5	57.3^*^	22
1–3 years at university/university college	28.8	53.9^*^	53
4 years or more at university/university college	28	55.5^*^	67
**Mothers' income**			
Up to 299 000 Norwegian kroner	32.2	54.6^*^	39
300 000–499 000 Norwegian kroner	32	57.7^*^	103
500 000–699 000 Norwegian kroner	25.8	54.4^*^	38
700 000–899 000 Norwegian kroner	19.6	48.9	3
900 000–999 000 Norwegian kroner	24.2	63.3	4
More than 1000 000 Norwegian kroner	34.2	56.4	5
**Mothers' age**			
20–24 years-old	34.2	57.7^*^	6
25–29 years-old	33	55.1^*^	41
30–34 years-old	31.8	59.8^*^	64
35–39 years-old	31.4	61.7^*^	49
40 years or older	26.2	57.9^*^	25

* *Significant higher MVPA at kindergarten time compared to leisure time, p < 0.05*.

## Results

The results in Table 3 reveal that 84% of the children reached the international health recommendations of physical activity of 60 MVPA daily during their weekdays and weekends, taking their total MVPA into account. In addition, only 3.7% of the children achieved the international health recommendations of physical activity of 60 MVPA daily on weekdays during their leisure, while 39.8% of the children reached the international health recommendations of physical activity of 60 MVPA daily on weekdays during their time in kindergarten. Further calculations showed that the time children spent at preschool contributed to 48.8% of the children's total MVPA.

**Table 3:** Descriptive characteristics of children (aged 4–6 years-old): Minutes in MVPA and fulfilling health recommendations.

	**Boys (SD)**	**Girls (SD)**	**Total (SD)**
Sample size (*n*)	125	119	244
MVPA preschool hours (minutes)	61.7 ± 18.3	55.1 ± 17.3	58.4 ± 18.1
MVPA leisure time weekdays (minutes)	33.6 ± 12.6	30.8 ± 12.8	32.3 ± 12.8
MVPA weekend (minutes)	75.6 ± 31.5	69.3 ± 27.9	72.5 ± 29.9
**Health recommendations**			
Met (%)	89.6	78.2	84
Met during preschool hours weekdays (%)	45.6	33.6	39.8
Met outside preschool hours weekdays (%)	5.6	1.7	3.7
Met during weekends (%)	0	0	0
Not met (%)	10.4	21.8	16

**Figure 1:**
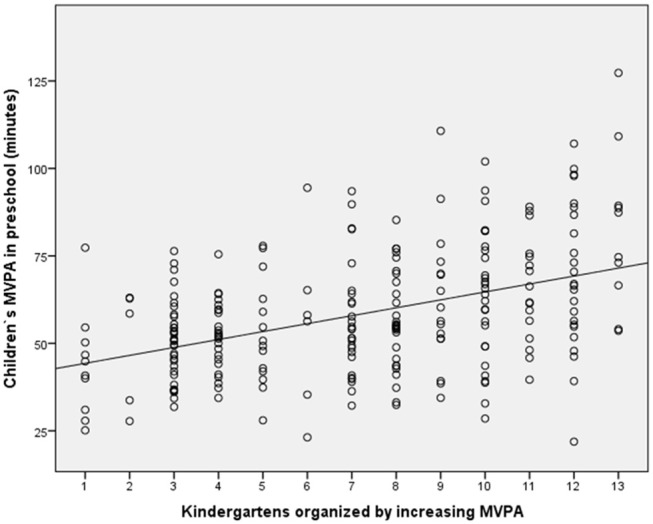
Scatter plot with preschool children's MVPA at the 13 kindergartens organized by increasing MVPA level on the X axis, and preschool children's MVPA at kindergarten on the Y axis.

A one-way ANOVA revealed that the pre-schoolers' MVPA level at kindergarten is significantly different between the 13 kindergartens (*F*_12_ = 5.1, *p* < 0.001). In Figure 1, the activity level of children at kindergarten is organized by increasing (mean) minutes of MVPA in the 13 kindergartens.

*post hoc* tests with Bonferroni corrections showed that kindergartens 12 and 13 had significantly higher activity levels than kindergartens 1–3 and 1–4, respectively (*p* < 0.05). The intraclass correlation revealed that 19.5% of the total variance in MVPA at kindergarten is explained by the kindergarten.

To elucidate how the kindergarten succeeded to reduce differences in activity level that could have arised at leisure time, related to their mothers' education level, income, and age (controlled for the effect of gender), was a main aim of the study. Table 2 present descriptive data concerning how these independent variables are related to preschool children's MVPA, both at kindergarten and leisure.

The results in Table 2 show that the children's activity level is significantly higher at kindergarten time than at leisure time on weekdays in almost all groups. The data indicate that neither mothers' education level, income or age seem to have linear associations with MVPA at leisure or at kindergarten. The results of the linear regression analyses that are presented in Table 4 identify which of the variables predict activity level at kindergarten.

**Table 4:** Factors associated with preschool children's MVPA level at kindergarten.

**Variables**	**Model 1 b (st.e.), *p***	**Model 2 b (st.e.), *p***	**Model 3 b (st.e.), *p***
Increasing MVPA kindergarten	−2.27 (0.31), *p* < 0.001	−2.57 (0.38), *p* < 0.001	−2.33 (0.34), *p* < 0.001
Mothers' education level (low/high)		−4.07 (2.84), *p* = 0.154	−2.61 (2.59), *p* = 0.314
Mothers' income (low/high)		−1.80 (3.23), *p* = 0.578	−0.23 (2.92), *p* = 0.937
Mothers' age		−0.45 (1.36), *p* = 0.739	0.14 (0.27), *p* = 0.612
MVPA leisure			0.59 (1.00), *p* < 0.001
Children's gender			−5.08 (2.32), *p* < 0.05
Constant/*R*^2^	73.77/0.19	86.88/0.20	58.82/0.36

**Figure 2:**
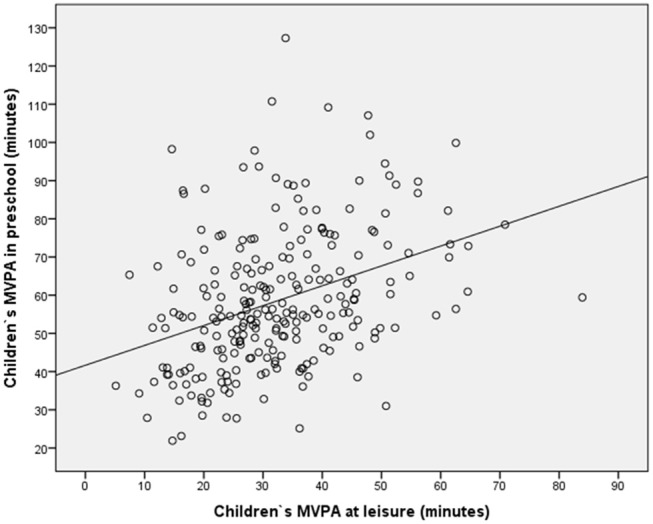
Scatter plot with preschool children's MVPA at leisure on weekdays on the X axis, and the same preschool children's MVPA at kindergarten on weekdays on the Y axis.

**Figure 3:**
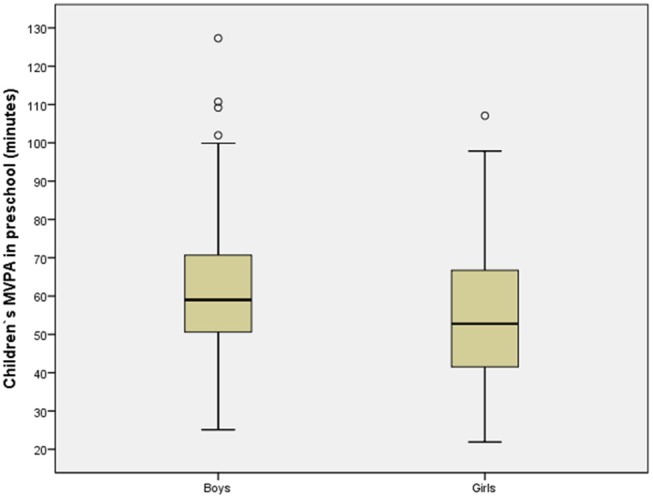
Scatter plot with boys and girls on the X axis, and their MVPA at kindergarten on the Y axis.

In model 1 the 13 kindergarten with increasing MVPA was included in the linear regression (Table 4). In model 2, mothers' education level, income, and age were also included, while children's gender and MVPA at leisure were included together with the other independent variables in model 3. The results in Table 4 reveal that neither mothers' education level, income, or age predicted MVPA at kindergarten (*p* > 0.05). Furthermore, the results also show that the effect of kindergarten was stable, controlling for mothers' education level, income, and age, and children's MVPA during leisure. However, Table 4 shows that children's MVPA during leisure and children's gender predicted MVPA in kindergarten. These findings are visualized in Figures 2, 3, respectively.

Figure 2 shows how the preschool children's MVPA at leisure is associated with their MVPA at kindergarten, and how the kindergarten succeeded to create high levels of MVPA at kindergarten for preschool children with low activity levels at leisure. The figure demonstrates that a positive association exists between MVPA at leisure and MVPA at kindergarten (a significant association, as shown in Model 3, Table 4), in which MVPA at kindergarten increases when MVPA at leisure increases. Figure 3 presents the MVPA level among girls and boys at kindergarten. It is shown that MVPA level among boys is higher than among girls at kindergarten.

## Discussion

The first main finding was that 84% of the children satisfied the international health recommendations for physical activity of 60 min of MVPA daily during weekdays and weekends, taking their total MVPA into account. Furthermore, the results showed that MVPA during kindergarten was the main contributor to preschool children's total MVPA during weekdays, by contributing approximately twice as much to MVPA level during weekdays than MVPA during leisure time—a significant difference (Table 2). During the entire week, preschool contributed to approximately half of the children's MVPA. This is in accordance with Finn et al. (14), who identified day care as the strongest determinant for physical activity. The activity level of 4–6 years old children in kindergarten time in our study (mean MVPA = 58.4) is somehow in accordance with other studies. Cauwenberghe et al. (45) examined children's activity in kindergarten time, finding that the average time spent on MVPA was 44.2 min on days with organized activity, and 34.1 min on days without organized activity. Dønnestad et al. (46) determined that 56% of children aged between three and six satisfied the recommendations of 60 min of daily physical activity in day care. In a study of 247 children aged three to five, Pate et al. (7) reported that these children were active in MVPA for 7 min an hour during their time in day care.

The second main finding was that MVPA levels were significantly different between the 13 kindergartens. Two of them stood out with significantly higher activity levels during kindergarten time than the three kindergartens with the lowest activity levels. The analyses showed that 19.5% of the total variance in MVPA at kindergarten can be attributed to the particular kindergarten. This is in accordance with Froberg and Bugge (28), who found in their study that children are influenced by the kindergarten that they attend, regarding how physically active they are. Our findings, that the differences in levels of activity are created in day care, and that no differences exist between children's MVPA out of day care, are supported by both O'Neill et al. (47) and Grøntvedt et al. (48). The fact that the differences in children's activity levels are not created in the children's spare time, but in kindergartens led by “professionals,” is a finding that is both surprising and problematic from a social perspective. Our findings point to the importance of striving for a culture in which the staff adapt to common values and nurture a collaborative culture for increasing physical activity (49). Our study may suggest that the three kindergartens that exhibit the lowest activity levels may need to emphasize work with physical activity to a greater extent than they currently do. Despite the culture of spending lot of time outdoors in Norwegian kindergartens, which boosts childrens physical activity level (23), some kindergartens do not manage to give all children sufficient physical activity in kindergarten. Bjørgen and Svendsen (26) identified the critical importance of kindergarten staff being involved and making efforts to promote children's physical activity, and highlights the importance of enthusiastic adults that initiate, lead, and are excited about the activities as the key to stimulation of motivation and enjoyment.

One of the main aims of the present study was to elucidate how the kindergarten succeeded to reduce differences in activity level that may arise at leisure time, related to their mothers' education level, income, and age. The results in Table 4 show that preschool children's MVPA level during kindergarten time was not associated with mothers' age, education level, or income. However, Table 2 show that childrens' MVPA level at leisure time was not related to their mothers' education level, income, or age. This finding may seem surprising in light of the extant literature. Borraccino et al. (25) found that the physical activity levels of children, increased with parents' socio-economic status. In a longitudinal study, Cleland et al. (50) found that, for boys, the mother was important as a role model for physical activity, and the father's reinforcement (praise for participation in physical activity) and direct support (bringing the child to activities, payment for participation and equipment) constituted the factors that influenced physical activity most positively. For the girls, it was the mother's active participation in physical activity, as well as that of siblings, that was most critical in relation to physical activity in MVPA. We will argue that our findings indicate that children are naturally active, and that the sociological processes leading to differences in activity level that have been found among adolescents, have not yet been elucidated. The children's sports provisions in Norway do not permit children younger than six to compete in sports (51), and participation in organized sport do not start before children start school. This may contribute to the fact that the focus on the amount and quality of physical activity begins when the children start school. The fact that children's level of physical activity is not associated with socio-economic status, may also be because children of kindergarten age have a natural need for movement. It is known that children require a shorter time for restitution regarding heart rate, ventilation, and CO_2_ than adolescents, and children's tempo in physical activity may be explained according to restitution time (52). We would point out, however, that our study is based on the socio-economic status of the children's mother. Furthermore, our results revealed that boys were more active than girls, resulting in more boys than girls meeting the health recommendations for physical activity. Thus, our findings support several studies which have found that boys are more active than girls (7, 9, 10, 14–17). However, it is worth noting that Pate et al. (7) asserted that boys being more active than girls may be linked to how the staff behave as role models for boys and girls in day care, as well as to what the staff think about the gender roles of boys and girls. Pate et al. (7) argues that the differences between girls and boys are not biological, but are rather due to socio-cultural factors. Typically, in physical activity, boys play in larger groups, with greater risk, and with more bodily contact. Pate also proposed that girls receive less encouragement to participate physically in the course of the day in day care. Penpraze et al. (17) also argued that the differences between girls and boys are not biological, citing that girls are more active during weekends than boys. Our study, on the other hand, does not find that girls are more active than boys during weekends (Table 3).

The fourth main finding was that a positive association existed between MVPA during leisure time and MVPA at kindergarten, in which MVPA at kindergarten increases when MVPA during leisure increases (Table 4). In other words, the kindergartens do not reduce inequality according to the physical activity level among preschool children that occurs during leisure time. In general, day care increases such differences and contributes to creating even larger differences between low-active and high-active children. O'Neill et al. (47) found that children who did not meet the PA guidelines in school, did not “catch up” with children who met the guidelines. This underlines the importance of increasing the level of physical activity for all children in day care. Kindergartens must adapt to the individual child's need for physical activity. Kindergartens are obliged to promote equal opportunities and equality, to base their activities on principles of equal rights and non-discrimination, and to facilitate the children to interact in, and create, an equal society (53). Overall, everyone must have equal opportunities to be seen, heard, and encouraged to participate together in all activities in day care. The General Plan for Norwegian kindergartens, establishes that the staff must reflect on their own attitudes to be able to optimally present and promote equality and equal rights according to physical activity level (53). However, the findings indicate that kindergartens do not manage to counterbalance the differences in MVPA during leisure time between preschool children with high and low activity levels during leisure time. On the other hand, kindergartens increase the differences according to the children's total MVPA.

### Strength and Limitations of the Study

The present study possesses several advantages. It includes a large number of participants, reflecting the actual gender distribution of boys and girls in Norwegian kindergartens. Different types and sizes of kindergartens were also included in the study, as a result of being randomly selected, which yielded a representative sample. To the best of our knowledge, this is the first study to objectively assess children's physical activity both at kindergarten and spare time with accelerometers, and at the same time examine the importance of kindergarten as a reducer of inequality according to the physical activity level of preschool children. Accelerometers, as an objective measurement, decrease subjectivity (54), and eliminate bias, such as social desirability, and recall problems (34). Furthermore, several researchers identified accelerometers as the optimal method to capture physical activity in free living situations (36, 55). The Actigraph GT1M is validated and reliability-tested for measuring physical activity levels for children aged 0–5 (56, 57), and against the international health recommendations (58). Nevertheless, the present study is not without limitations. The use of questionnaires in order to describe the education level and income of the children's mothers, which were dichotomized into categorical variables, only examines the association of high and low income and education level. Data about the mothers' income and education at an interval level would have been preferable. Moreover, although accelerometery is considered to be an optimal measurement when assessing physical activity in free-living situations, it underestimates activities related to cycling or riding vehicles (54), which is especially unfortunate, as riding vehicles has been argued to be important for pre-schoolers' physical activity (59). Furthermore, neither swimming nor other water activities (due to the instruction of no water-contact) were included in the data analysis, which might lead to an error estimation of the children's physical activity level.

## Conclusion

The results show that MVPA during kindergarten time is the main contributor to preschool children's total MVPA during weekdays, by contributing approximately twice as much to the MVPA level on weekdays than MVPA at leisure on weekdays. Furthermore, boys were more active than girls, and preschool children's MVPA level at both leisure and at kindergarten were not associated with either mothers' age, education level, or income. On the other hand, a positive association was found between MVPA at leisure and MVPA at kindergarten, in which MVPA at kindergarten increases when MVPA at leisure increases. Moreover, MVPA levels were significantly different between kindergartens, in which two of the 13 kindergartens stood out with significantly higher activity levels at kindergarten time than the three kindergartens with the lowest activity levels. To our surprise, the results indicated there were no differences in MVPA at leisure between children from different kindergartens, but rather that the kindergartens themselves created such differences—contributing to create differences according to low-active and high-active children.

It is difficult to state with certainty which factors lead to different activity levels from one kindergarten to the next. Consequently, further research should more closely examine what distinguishes those with high activity levels from those with lower activity levels in terms of staff culture.

## Ethics Statement

The subjects were fully informed about the protocol prior to participating in the study. A written consent form was signed by the parents of the children, according to accepted ethical research regulations. Approval to use the data and conduct the study was given by the Norwegian Social Science Data Services (NSD).

## Author Contributions

KK has contributed on design and methods, writing the introduction, discussion, and conclusion. Furthermore, a critical review of all the text during several numbers of the article and rewriting of the text. PL has contributed on design and methods, writing the introduction, methods, discussion, and the conclusion.

### Conflict of Interest Statement

The authors declare that the research was conducted in the absence of any commercial or financial relationships that could be construed as a potential conflict of interest.
